# Knowledge about hepatitis B and hepatitis C virus infection and consequences: a cross-sectional assessment of baseline knowledge among infected patients in West Bengal, India

**DOI:** 10.1186/s41124-016-0014-8

**Published:** 2016-08-08

**Authors:** Partha Sarathi Mukherjee, Eliza Dutta, Dipesh Kr. Das, Shatabdi Ghosh, Suvadip Neogi, Arka Sarkar

**Affiliations:** Liver Foundation, West Bengal, 12, Kyd Street, Kolkata, 7000016 India

**Keywords:** HBV, HCV, Patients, Knowledge, Awareness

## Abstract

**Background:**

India has a high burden of disease from hepatitis B virus (HBV), with 3.7 % point-prevalence, as well as from hepatitis C virus (HCV), with 1–1.5 % prevalence. Societal ignorance about HBV and HCV in India limits the potential for prevention and treatment efforts to bring these diseases under control. Since patients’ own knowledge about their health condition may have important health consequences, this study sought to assess knowledge levels among HBV and HCV patients referred to the virology laboratory of the Liver Foundation, West Bengal.

**Methods:**

Patients who had tested positive for HBsAg or anti-HCV at government specialty clinics were invited to enroll in the study when they presented for follow-up laboratory testing. Study participants completed a survey that contained three multiple-choice questions about viral hepatitis etiology and five multiple-choice questions about the consequences of HBV and HCV infection. Mean knowledge scores for male and female respondents were compared, and comparisons were also made across different places of residence, age groups, education levels and income levels. One-way ANOVA was used to test for significant differences.

**Results:**

Among 520 study participants, the mean knowledge score was 4.76 on an eight-point scale. Approximately 40 % of the study sample scored less than 4.0. Almost three-quarters of respondents correctly responded to the question, “Which organ of the human body is affected by hepatitis?” while almost two-thirds knew how hepatitis B is transmitted. Regarding consequences of HBV and HCV infection, less than one-third of study participants answered correctly when asked, “What happens when one is infected with hepatitis B or C?” Slightly more than two-thirds of people correctly answered the question about how hepatitis B is prevented. The mean knowledge score varied across age groups (*P* = 0.0009), education levels (*P* = 0.0001) and monthly household income levels (*P* = 0.0001). With higher levels of schooling and higher household income, there were corresponding increases in knowledge scores.

**Conclusion:**

There is room for improving knowledge of HBV and HCV etiology and consequences among patients as well as healthcare workers in India. More awareness activities should be organized, accompanied by further research to track whether knowledge scores improve over time.

## Background

Viral hepatitis has emerged as a major health problem worldwide, including in India. Among the five main types of hepatitis viruses, hepatitis B virus (HBV) and hepatitis C virus (HCV) are of the greatest concern due to their burden of illness and death. HBV and HCV can cause both acute and chronic disease [1, 2]. An estimated 240 million people are chronically infected with HBV (defined as hepatitis B surface antigen-positive for at least 6 months) and more than 780,000 people die every year due to complications of HBV, including cirrhosis and liver cancer [1, 3–7]. Between 130 and 150 million people globally are thought to have chronic HCV infection. As with HBV, potential outcomes of chronic HCV infection include liver cirrhosis and liver cancer. Approximately 500,000 people die each year due to HCV-related liver ailments [2, 7].

An estimated 100 million chronic HBV and HCV carriers reside in Southeast Asia [8–10]. Home to almost one-fifth of the world’s population, India accounts for a large proportion of the global HBV burden, with 10–15 % of the entire pool of HBV carriers. It is considered an intermediate endemic country, with a 3.7 % point-prevalence [11, 12]. More than 40 million HBV carriers live there; annually, one million Indians are at risk of getting HBV infection, and about 100,000 deaths occur due to HBV infection [3, 5]. Approximately one million infants in India live with the lifetime risk of developing chronic HBV infection [11, 13]. Due to its large population, the country also accounts for a significant share of the global HCV burden, with 1–1.5 % prevalence. An estimated 15 to 18 million people live with acute or chronic HCV infection in India [14, 15]. About 20 % of liver diseases in the country are associated with HCV infection, and HBV infection accounts for an even larger proportion of the burden of liver diseases [8].

HBV and HCV are bloodborne diseases, and knowledge about transmission pathways and preventive measures can help reduce the risk of acquiring both diseases. Furthermore, appropriate medical treatment can lessen the effects of HBV and can cure HCV. However, societal ignorance in India about HBV and HCV limits the potential for prevention and treatment efforts to bring both epidemics under control [16].

Low knowledge about HBV and HCV may have serious consequences for patients and their families and other contacts. Patients who lack adequate information or are misinformed about HBV and HCV may not be sufficiently prepared to make decisions that can protect their health, including adhering to prescribed medical treatments. Patients’ poor knowledge might also lead them unknowingly to expose their family members and others to HBV or HCV. Therefore, dissemination of correct information to patients as part of a holistic approach to their health care might help them develop good disease management strategies and might also constitute an essential component of effective viral hepatitis prevention programs, ultimately helping to reduce the burden of disease and death in India.

Additional negative consequences might arise from prevailing misconceptions about HBV and HCV, according to field experiences reported by the Liver Foundation, West Bengal (LFWB), an Indian nongovernmental organization. These misconceptions may deter people from seeking proper medical advice and may prompt some of them to turn to folk medicines for treatment. In the case of HIV, patients’ limited knowledge and wrong perceptions about that disease have been linked to poor treatment outcomes [17]. This finding raises the question of whether a similar dynamic may undermine efforts to treat HBV and HCV patients. Furthermore, these patients may experience shame associated with the stigmatized nature of these diseases, potentially leaving them isolated and afraid to seek appropriate medical care, particularly those with limited knowledge about their health. Knowledge of the etiology and consequences of HBV and HCV may thus empower them to take an active part in their own treatment.

The present study was undertaken to assess HBV- and HCV-infected Indian patients’ knowledge regarding HBV and HCV etiology and consequences. The majority of studies assessing knowledge, attitudes and practices regarding HBV and HCV in low- and middle-income countries have been conducted among medical and laboratory technicians [18–21], while a few have focused specifically on HBV and HCV knowledge among the general population [22, 23]. To the best of our awareness, this is the first study to investigate Indian HBV and HCV patients’ knowledge of the etiology and consequences of HBV and HCV infection. The purpose of the study was to acquire evidence that can help to inform the future direction of HBV and HCV control efforts in the study location, West Bengal, and in other settings with similar populations and similar disease control objectives.

## Methods

### Setting and participants

The Liver Foundation, West Bengal works to raise awareness about HBV and HCV, liver disease and general health issues. Since 2007, LFWB has been conducting awareness and health education meetings in schools, colleges and community settings. In addition to the general population, target audiences include patients, physicians and other healthcare workers.

With the support of the Bristol-Myers Squibb Foundation (BMSF), LFWB established a laboratory to provide low-cost molecular virology and metabolic diagnosis for patients with liver diseases. Patients typically make their first visit to the LFWB laboratory following a two-stage diagnostic process. When a general physician has diagnosed a patient with a liver problem, it is standard practice for the patient to be referred to the speciality clinic of a government hospital. A consultant at the specialty clinic may advise the patient to undergo serological testing for HBsAg and anti-HCV, and patients who do so are tested at the same clinic. Those patients who test positive for either disease marker are then referred to the LFWB molecular virology laboratory for further testing.

For the purpose of this study, HBsAg-positive patients referred to the LFWB laboratory from government hospitals were considered by investigators to have a diagnosis of HBV, and similarly, anti-HCV-positive patients were considered to have a diagnosis of HCV. Patients with a diagnosis of HBV or HCV were invited to participate in the study at the time of their visit to the LFWB laboratory if they also met the following study inclusion criteria: (1) the patient was at least 18 years old, (2) the patient had previously undergone no more than one liver-related consultation with a certified physician at a government hospital, and (3) the patient was visiting the LFWB laboratory for the first time.

Patients who met these criteria and agreed to participate in the study were asked to immediately complete a study survey at the central office of the LFWB, which is adjacent to the laboratory. Study enrollment and data collection took place between February 2014 and January 2015. To ensure that study participants fully comprehended all survey questions, study investigators verbally administered the survey instrument in face-to-face interviews and explained questions if required to do so. The interviews were conducted in participants’ native languages. Although Hindi and English were used in some cases, Bengali, or “Bangla,” was used more often as it is the native language of the province (West Bengal).

### Survey instrument

Study data were collected using a two-part questionnaire developed by investigators. The survey instrument was validated by administering it to 20 randomly selected HBV or HCV patients who fulfilled the study criteria. In the first part of the survey instrument, the following patient profile information was collected: name, age, sex, address, education background and monthly household income. The second part of the survey instrument was composed of eight multiple-choice questions: three about viral hepatitis etiology and five about the consequences of HBV and HCV infection. There were four multiple-choice options for each question, among which one was correct (Table 1).

**Table 1:** Questions put to study participants about viral hepatitis

1. Which organ of the human body is affected by hepatitis? a. Kidney b. Liver c. Stomach d. Do not know 2. Which is the most severe disease affecting the liver? a. Jaundice b. Hepatitis B and C c. Hepatitis A and E d. Do not know 3. How is hepatitis B or C transmitted? a. Through contaminated food and water b. Through sneezing and coughing c. Through contaminated blood d. Do not know 4. What happens when one is infected with hepatitis B or C? a. Jaundice will occur b. Jaundice may not occur c. Jaundice will never occur d. Do not know 5. How is jaundice cured? a. Wear “garland” of jaundice b. Take complete bed rest c. Take only boiled food d. Take proper medical advice 6. What should be the diet during jaundice? a. Completely vegetarian food b. Completely boiled food (without oil and spices) c. Glucose, sugarcane juice and fruits d. Normal healthy diet (homemade) 7. What should one do when affected with jaundice? a. Take leave from job and have complete bed rest b. Do normal work c. Do excess work d. Do not know 8. How is hepatitis B or C prevented? a. By isolating the infected person b. By avoiding street food c. Through vaccination and proper advice d. Do not know	

Several questions used the term “jaundice” instead of asking directly about HBV and HCV because most of the lay public in India, even paramedics, think of jaundice as a liver disease while not being aware of viral hepatitis.

Moreover, the questionnaire was categorized into two parts, an etiology section with fewer questions and a consequences section with more questions, as the survey was conducted among infected patients who had had one specialist consultation.

### Scoring

When a respondent chose either an incorrect response, multiple responses or “do not know,” the question was scored as nil. Each correct response was given a score of 1. A respondent’s total score thus could range from 0 to 8.

### Statistical analysis

Knowledge scores were calculated for individual study participants, and descriptive statistics were generated from these data. Knowledge scores then were considered in relation to five sociodemographic factors: place of residence, sex, age, education background and monthly household income. For place of residence, mean knowledge scores were compared across 19 districts and one other area. For sex, male and female study respondent scores were compared. Other comparisons were made using five age groups, five education levels and five income levels. One-way ANOVA was used to test for significant differences between mean knowledge scores for the categories associated with each sociodemographic factor [24].

### Ethics

Participation in this study was voluntary. Written consent was obtained from all participants prior to study enrollment.

## Results

Among 603 patients invited to participate in the study, 520 agreed and completed surveys, for a response rate of 86.2 %. Respondents resided in 19 districts in West Bengal, as well as in two neighboring states, Jharkhand and Bihar, and one neighboring country, Bangladesh. However, more than half were from three districts: Kolkata (23.5), North 24 Parganas (14.0) and South 24 Parganas (15.8 %) (Table 2). The study population was composed of 342 males and 178 females. The overall mean age was 36.7 years, ranging between 18 and 75 years of age (data not shown). Forty-two percent of study participants reported that the highest education level they had completed was middle school, while 11.7 of study participants had completed high school and 24.1 % had completed college or university. Almost 60 % of study participants belonged to households earning less than 5546 INR (approximately 81 USD) per month.

**Table 2:** Sociodemographic characteristics of the study population and mean knowledge scores

Variable	(%) N	Mean score on 8-point scale	*P*-value
District (*n* = 520)			
Bankura	1.5 (8)	3.50	0.122
Birbhum	2.3 (12)	4.92	
Burdwan	3.3 (17)	4.12	
Coochbehar	0.2 (1)	4.00	
Darjiling	0.2 (1)	4.00	
East Midnapore	4.2 (22)	5.45	
Hooghly	6.0 (31)	5.06	
Howrah	7.5 (39)	4.38	
Jalpaiguri	0.2 (1)	6.00	
Kolkata	23.5 (122)	5.14	
Malda	2.9 (15)	4.87	
Murshidabad	6.3 (33)	4.70	
Nadia	7.1 (37)	4.84	
North 24 Pargana	14.0 (73)	5.14	
North Dinajpur	0.6 (3)	3.00	
Purulia	0.4 (2)	4.50	
South 24 Pargana	15.8 (82)	4.02	
South Dinajpur	0.8 (4)	5.25	
West Midnapore	1.9 (10)	4.20	
Other	1.4 (7)	5.43	
Sex (*n* = 520)			
Male	65.8 (342)	4.73	0.574
Female	34.2 (178)	4.84	
Age (years) (*n* = 520)			
< 25	15.4 (80)	4.70	0.009
25 – 34	34.2 (178)	5.04	
35 – 44	23.7 (123)	4.98	
45 – 54	15.2 (79)	4.44	
55+	11.5 (60)	3.98	
Education (*n* = 489)^a^			
No formal education^b^	3.1 (15)	2.80	0.0001
Completed primary education (4th standard)	19.6 (96)	3.56	
Completed middle school (8th standard)	41.5 (203)	4.78	
Completed high school (12th standard)	11.7 (57)	5.42	
Completed college/university or above	24.1 (118)	6.18	
Total monthly household income (INR) (*n* = 399)^c^			
Less than 1865	17.3 (30)	3.57	0.0001
1866 – 5546	40.6 (203)	4.48	
5547 – 9248	25.3 (68)	5.13	
9249 – 13 873	5.3 (39)	5.41	
More than 13 873	11.5 (59)	6.10	

^a^31 respondents did not answer the question about education

^b^Respondents who reported that they had received less than 4th standard education were classified as “no formal education.”

^c^121 respondents did not answer the question about income

Table 3 shows the proportions of study participants providing correct, incorrect and “do not know” responses to the eight questionnaire items. Almost three-quarters of respondents correctly responded to the question, “Which organ of the human body is affected by hepatitis?” while almost two-thirds knew how hepatitis B is transmitted. Forty-six percent replied correctly when asked, “Which is the most severe disease affecting the liver?” Regarding consequences of HBV and HCV infection, less than one-third of study participants answered correctly when asked, “What happens when one is infected with hepatitis B or C?” Much larger proportions of respondents replied correctly when they were asked, “How is jaundice cured?” (72.1) and “What should be the diet during jaundice?” (61.9 %). Slightly more than two-thirds of participants correctly answered the question about how hepatitis B or hepatitis C is prevented.

**Table 3:** Proportions of correct and incorrect responses on eight-item questionnaire (*N* = 520)

Question	Responses (%)		
	Correct	Incorrect	Do not know
Etiology			
Which organ of the human body is affected by hepatitis?	74.2	1.9	23.8
Which is the most severe disease affecting the liver?	46.0	20.8	33.3
How is hepatitis B or C transmitted?	63.5	8.1	28.5
Consequences			
What happens when one is infected with hepatitis B or C?	32.7	40.4	26.9
How is jaundice cured?	72.1	23.3	4.6
What should be the diet during jaundice?	61.9	33.8	4.2
What should one do when affected with jaundice?	58.1	33.7	8.3
How is hepatitis B or C prevented?	67.9	10.8	21.3

The mean knowledge score reported in this study was 4.76 on an eight-point scale. Approximately 40 % of the study sample scored less than 4.0 (only half of the possible points). The mean score varied significantly across age groups (*P* = 0.009), with people aged 25–34 years scoring the highest (5.04) and people aged 55 years and older scoring the lowest (3.98). Mean scores also varied significantly across education levels (*P* = 0.0001) and monthly household income levels (*P* = 0.0001), increasing with higher education as well as higher income (Table 2).

When knowledge scores were disaggregated into etiology and consequences, significant differences were observed in both education (*P* = 0.0001) and household income (*P* = 0.0001) domains (Table 4). At higher levels of schooling and household income, knowledge scores in both domains improved. Regarding the relationship between knowledge scores and age, significant variation was found for knowledge about consequences of HBV and HCV infection (*p* = 0.0001) but not for knowledge about etiology. The highest knowledge score for the five “consequences” questions was 3.17 among people aged 35–44 years, while the lowest knowledge score was 2.28 among people aged 55 years and older.

**Table 4:** Mean scores for knowledge of etiology and consequenses of hepatitis B and C, disaggregated by sociodemographic factors

	Etiology Max score = 3 (95 % confidence interval)	*P* value	Consequences Max score = 5 (95 % confidence interval)	*P* value
District (*n* = 520)				
Bankura (*n* = 8)	1.25 (0.78 – 2.32)	0.517	2.25 (0.72 – 3.78)	0.089
Birbhum (*n* = 12)	2.00 (1.19 – 2.81)		2.92 (1.92 – 3.91)	
Burdwan (*n* = 17)	1.59 (1.01 – 2.16)		2.53 (1.78 – 3.28)	
Coochbehar (*n* = 1)	2.00		2.00	
Darjiling (*n* = 1)	2.00		2.00	
East Midnapore (*n* = 22)	2.00 (1.51 – 2.49)		3.45 (2.83 – 4.08)	
Hooghly (*n* = 31)	1.84 (1.45 – 2.23)		3.22 (2.76 – 3.69)	
Howrah (*n* = 39)	1.82 (1.49 – 2.14)		2.56 (2.056 – 3.07)	
Jalpaiguri (*n* = 1)	2.00		2.00	
Kolkata (*n* = 122)	1.99 (1.79 – 2.18)		3.14 (2.89 – 3.41)	
Malda (*n* = 15)	2.07 (1.49 – 2.64)		2.81 (2.01 – 3.59)	
Murshidabad (*n* = 33)	1.79 (1.37 – 2.21)		2.91 (2.43 – 3.38)	
Nadia (*n* = 37)	1.76 (1.38 – 2.13)		3.08 (2.58 – 3.58)	
North 24 Pargana (*n* = 73)	1.99 (1.74 – 2.23)		3.15 (2.88 – 3.42)	
North Dinajpur (*n* = 4)	1.00 (−1.48 – 3.48)		2.00 (−2.97 – 6.87)	
Purulia (*n* = 2)	2.00 (−10.71 – 14.71)		2.500 (−16.56 – 21.56)	
South 24 Pargana (*n* = 82)	1.54 (1.28 – 1.79)		2.49 (2.18 – 2.79)	
South Dinajpur (*n* = 4)	2.25 (0.73 – 3.77)		300 (1.16 – 4.84)	
West Midnapore (*n* = 10)	1.60 (0.99 – 2.20)		2.60 (1.91 – 3.29)	
Other (*n* = 7)	2.14 (1.02 – 3.27)		3.29 (2.13 – 4.45)	
Sex (*n* = 520)				
Male (*n* = 342)	1.79 (1.67 – 1.91)	0.200	2.93 (2.78 – 3.08)	0.895
Female (*n* = 178)	1.92 (1.76 – 2.08)		2.92 (2.71 – 3.12)	
Age (years) (*n* = 520)				
< 25 (*n* = 80)	2.02 (1.79 – 2.26)	0.151	2.68 (2.36 – 2.99)	0.0001
25 – 34 (*n* = 178)	1.90 (1.75 – 2.05)		3.14 (2.94 – 3.33)	
35 – 44 (*n* = 123)	1.81 (1.62 – 2.00)		3.17 (2.91 – 3.43)	
45 – 54 (*n* = 79)	1.63 (1.37 – 1.89)		2.81 (2.53 – 3.09)	
55+ (*n* = 60)	1.70 (1.38 – 2.02)		2.28 (1.89 – 2.68)	
Education (*n* = 489)^a^				
No formal education^b^	0.93 (0.29 – 1.58)	0.0001	1.87 (1.24 – 2.49)	0.0001
Completed primary education (4th standard)	1.31 (1.08 – 1.54)		2.25 (1.99 – 2.50)	
Completed middle school (8th standard)	1.79 (1.64 – 1.94)		2.99 (2.79 – 3.18)	
Completed high school (12th standard)	2.21 (1.95 – 2.47)		3.21 (2.93 – 3.49)	
Completed college/university or above	2.49 (2.37 – 2.61)		3.69 (3.49 – 3.89)	
Total monthly household income (INR) (*n* = 399)^c^				
Less than 1865 (*n* = 30)	1.53 (1.05 – 2.01)	0.0001	2.03 (1.51- 2.56)	0.0001
1866 – 5546 (*n* = 203)	1.61 (1.46 – 1.76)		2.87 (2.67 – 3.06)	
5547 – 9248 (*n* = 68)	1.99 (1.73 – 2.24)		3.15 (2.85 – 3.45)	
9249 – 13 873 (*n* = 39)	2.15 (1.88 – 2.43)		3.26 (2.86 – 3.65)	
More than 13 873 (*n* = 59)	2.36 (2.12 – 2.59)		3.75 (3.44 – 4.05)	

^a^31 respondents did not answer the question about education

^b^Respondents who reported that they had received less than 4th standard education were classified as “no formal education”

^c^121 respondents did not answer the question about income

## Discussion

The purpose of this study was to investigate knowledge of HBV and HCV etiology and consequences among infected patients in West Bengal, India. The study population of 342 males and 178 females included a large proportion of people who had a high school education or less, and household income levels reported by study participants were fairly low overall. The mean knowledge score for all study participants was 4.76 on an eight-point scale. Although significantly different knowledge scores were seen across age groups, disaggregating the “etiology” and “consequences” questions indicated that age groups differed significantly only in regard to knowledge of HBV/HCV consequences. Positive correlations were seen between HBV/HCV knowledge scores and education levels, as well as between these scores and household income levels.

Studies that have examined the association of socioeconomic status with knowledge of lung cancer and knowledge of stroke suggest a positive relationship [25–27]. Another study showed that lower socioeconomic status was associated with lower awareness about the harmful effects of smoking [28]. The present study found similar results: lower monthly household income correlated with less knowledge of viral hepatitis etiology and disease consequences, and knowledge scores increased progressively across higher income strata. The same pattern was found for educational attainment, which is widely regarded as a proxy for socioeconomic status [29]. We speculate that study participants with lower socioeconomic status might have had less access to information about HBV and HCV, including fewer opportunities to visit healthcare facilities to obtain such information. Costs of medical care, distance from such facilities and a lack of transportation might be related reasons for low knowledge among these patients, who might benefit from health policies and programs that consider these factors when developing HBV and HCV awareness campaigns.

If patients from lower socioeconomic strata are more inclined than other patients to utilize traditional healing practices, this may also help to explain the differences in knowledge scores, since traditional healing practices have the potential to foster misconceptions about HBV and HCV. We observed that 15 % of study participants who responded incorrectly to a question about a cure for jaundice believed in such local folk remedies as wearing a “jaundice-garland” to cure it. Twenty-one percent of all respondents believed that glucose in either a commercially available powder form or in the form of sugar cane should be the only diet of a person with jaundice. More research is needed to determine whether such beliefs and practices negatively affect HBV and HCV patients’ health outcomes, and to identify interventions that may improve the situation. There may be valuable guidance in other health fields; for example, research has been conducted on initiatives to train traditional healers in diabetes prevention and care in Cameroon, and to involve traditional healers in the scale-up of antiretroviral treatment for people living with HIV in Lesotho [30, 31].

With respect to age, we found that study participants aged 25–34 years had the highest overall mean knowledge score, and that study participants aged 35–44 years had the highest knowledge scores for HBV/HCV consequences, while those aged 55 years and older scored the lowest in both cases. The literacy rate in India increased from 12 % at the end of British rule in 1947 to 74 % in 2011, [32] which might partially explain our age-related study findings. However, there is still the question of why knowledge scores among study participants aged 18–24 years were also fairly low. There is a need for research that elucidates the relationship between age and HBV/HCV knowledge in India, as well as research that identifies key knowledge pathways for different age groups.

Provider-related factors should also be considered in relation to study participants’ low overall knowledge scores. All study participants were newly diagnosed with HBV or HCV, and all of them had received only one consultation related to the diagnosis. The consultation might not have been sufficient to provide a patient with adequate HBV or HCV knowledge. Previous studies have reported that patients’ inability to understand medical jargon, their fear of asking a medical practitioner for explanations about terminology, inadequate consultation time to address all queries due to over-burdened schedules, unfriendly healthcare staff and hospitality issues, patients’ fears of verbal abuse by physicians and allied healthcare staff, and their anxiety about and fear of the disease are major barriers to effective communication between patients and healthcare staff [33–35]. These barriers could be presumed to affect patients’ knowledge, potentially leading to misconceptions related to bloodborne viral hepatitis and increasing their health risks. A comprehensive disease prevention program should take such considerations into account.

Poor knowledge and misconceptions about HBV and HCV among general practitioners and other health workers might be another reason for low patient knowledge. Low levels of knowledge of bloodborne viral hepatitis among healthcare workers and medical students have been reported in studies conducted in developing countries. Setia et al., for instance, reported that 31 of medical practitioners, 38 of dental practitioners and 49 % of nurses claimed to have been vaccinated against hepatitis C, although no such vaccine exists [36]. Another study, conducted among students in four medical colleges in Pakistan, revealed that 73 % did not get screened for bloodborne hepatitis following a needlestick injury [37]. Studies have furthermore reported that discrimination and ostracism have originated from misinformation disseminated by healthcare personnel [38–41]. Informed doctors as well as other healthcare workers can play an important role in promoting accurate information about hepatitis and jaundice to patients. In doing so, health providers might also help reduce stigma for HBV and HCV patients across all settings, including operating rooms.

We suggest that these and other studies support the need to improve patients’ hepatitis knowledge through more effective HBV and HCV education programs targeting patients, healthcare practitioners and the general population alike. Such programs are particularly important in a country such as India, where jaundice is reported as a disease along with hepatitis in the National Sample Survey report and it is common for general practitioners to address jaundice instead of hepatitis as a disease, resulting in a society where yellowing of the eyes and urine is equated with jaundice as a synonym for hepatitis [42]. The National Program for Prevention and Control of Viral Hepatitis in India is not in action, even now, only a committee that was formed in 2012 to formulate such a control program in viral hepatitis [43]. National Family Health Survey, India reports reveal that liver disease, including hepatitis, is not a national public health priority; therefore more clarity in thought is needed from policy-makers [44].

A 2013 review concluded that educational interventions have significant beneficial effects on persons who are at risk for or have been diagnosed with HBV or HCV. These effects extend to disease knowledge and behavior modification, including getting testing for the disease, receiving vaccinations, and being willing to commence and adhere to treatment [45]. It is recognized that there is limited knowledge about infectious diseases in general, including viral hepatitis, particularly among people with a low educational and socioeconomic level. Therefore, more current information locally can lead to focused action for knowledge empowerment,which is also mentioned in the World Health Organization’s first-ever Global Health Sector Strategy on Viral Hepatitis [46]. Hence assessments of HBV and HCV patients’ knowledge levels are the necessary starting point in the process of determining how to overcome knowledge-related barriers to treatment and the prevention of onward transmission. Our present study offers important insights in this regard, and is notable for not being preceded by any other published research of this kind relating to an Indian population. Further research is needed, as the full population of HBV and HCV patients in India is diverse, with patients representing a wide range of cultural, socioeconomic and other backgrounds that may inform their knowledge and perspectives regarding viral hepatitis.

Some of the limitations of this study may have implications for the generalizability of its findings. More than half of the individuals who made up the relatively small sample of 520 reported that they lived in the districts of Kolkata, North 24 Pargana and South 24 Pargana. The heavy representation of these districts may be related to the large number of referrals that the LFWB receives, mainly from speciality clinics of government hospitals in Kolkata. Furthermore, the study did not include many participants from higher-income households. Almost 60 % of study participants reported having a monthly household income under 5547 INR, while monthly per capita income for West Bengal is INR 6575, and the national monthly per capita income is INR 7378 [47]. A larger study population, more evenly distributed across districts might have contributed to a better understanding of knowledge among HBV and HCV patients.

## Conclusion

The knowledge gaps observed among HBV and HCV patients in this study suggest a need for further research and for educational interventions that take into account the needs of diverse populations in India, including members of different age groups and socioeconomic strata. A country-wide focus on correct knowledge dissemination by healthcare professionals, improved physician-patient communication and the introduction of a positive health culture based on scientifically proven evidence can contribute to sustained viral hepatitis control in India.
